# Regulation of human and mouse telomerase genes by genomic contexts and transcription factors during embryonic stem cell differentiation

**DOI:** 10.1038/s41598-017-16764-w

**Published:** 2017-11-27

**Authors:** De Cheng, Shuwen Wang, Wenwen Jia, Yuanjun Zhao, Fan Zhang, Jiuhong Kang, Jiyue Zhu

**Affiliations:** 1 Department of Pharmaceutical Sciences, Washington State University College of Pharmacy, Spokane, Washington, USA; 2 School of Life Sciences and Technology, Tongji University, Shanghai, 200092 P.R. China; 3 Department of C & M Physiology, Pennsylvania State University College of Medicine, Hershey, Pennsylvania USA

## Abstract

Differential regulation of telomerase reverse transcriptase (TERT) genes contribute to distinct aging and tumorigenic processes in humans and mice. To study TERT regulation, we generated mouse embryonic stem cell (ESC) lines containing single-copy bacterial artificial chromosome (BAC) reporters, covering *hTERT* and *mTERT* genes and their neighboring loci, via recombinase-mediated BAC targeting. ESC lines with chimeric BACs, in which two TERT promoters were swapped, were also generated. Using these chromatinized BACs, we showed that hTERT silencing during differentiation to embryoid bodies (EBs) and to fibroblast-like cells was driven by the human-specific genomic context and accompanied by increases of repressive epigenetic marks, H3K9me3 and H3K27me3, near its promoter. Conversely, the mouse genomic context did not repress TERT transcription until late during differentiation. The hTERT promoter was more active than its mouse counterpart when compared in the same genomic contexts. Mutations of E-box and E2F consensus sites at the promoter had little effect on hTERT transcription in ESCs. However, the mutant promoters were rapidly silenced upon EB differentiation, indicating that transcription factors (TFs) bound to these sites were critical in maintaining hTERT transcription during differentiation. Together, our study revealed a dynamic hTERT regulation by chromatin environment and promoter-bound TFs during ESC differentiation.

## Introduction

Telomeres are protective caps of chromosomal ends that are critical for genomic stability^1^. Telomere DNA sequences are replenished by telomerase^2^, a reverse transcriptase complex containing a catalytic subunit (TERT), an RNA template (TERC), and accessory proteins^3,4^. Without telomerase, telomeres shorten upon successive cell divisions, leading to cell death or senescence^5,6^. Mutations in human telomerase genes (*hTERT*, *hTERC*) lead to dyskeratosis congenita, a multi-system disorder with a broad spectrum of clinical manifestations^7^. Moreover, ectopic hTERT expression in many cell types resulted in telomere elongation and cellular immortalization, indicating that hTERT is a limiting component of telomerase in human cells^8–10^.

Telomerase is highly expressed during early embryonic development, as well as in pluripotent stem cells, such as embryonic stem cells and germline cells^11–13^. In humans, while alternative mRNA splicing and post-translational modifications play roles in regulating hTERT expression^12^, the dominant event of hTERT regulation is its repression during development and differentiation^14^. Indeed, the level of hTERT mRNA is very low in most somatic tissues, with the exception of testis, ovary, thymus, and skin^15^. On the other hand, the *mTERT* gene is widely expressed in the majority of adult mouse tissues^16^. This broad expression is accompanied by very long telomeres in mouse cells and tissues^17^. Consequently, proliferative senescence of human cells, but not mouse cells, is telomere-dependent.

hTERT transcription is a primary step of telomerase regulation in many cell types^18^. To study its regulation in a relevant genomic context, we initially constructed BAC reporter H(wt), which contained a 160-kb human genomic sequence encompassing the entire *hTERT* locus, and its upstream and downstream neighboring loci, *CRR9* (also called *CLPTMIL* gene), and *Xtrp2* (or *SLC6A18*) loci. Previously, we showed that the hTERT promoter in randomly integrated H(wt) was highly active in mouse embryonic stem cells, and was efficiently repressed upon *in vitro* differentiation^13,19^. This was in contrast with the endogenous mTERT mRNA, which was only moderately down regulated during the same differentiation process and widely expressed in mouse tissues, indicating the significant differences in the regulation of *TERT* genes in human and mice^16^.

To study the species-specific TERT regulation during differentiation, we developed a new recombinase-mediated BAC targeting (RMBT) protocol to integrate H(wt) and M(wt), a BAC reporter containing a mouse genomic region equivalent to that in H(wt), into a chromosomal acceptor site in mouse ESCs. We showed that the hTERT promoter in H(wt), but not the mTERT promoter in M(wt), was progressively down-regulated during EB differentiation and further silenced upon differentiation into osteogenic and fibroblast-like cells. Furthermore, by using chimera BACs, in which the TERT promoters were swapped between H(wt) and M(wt), we showed that the repression of *hTERT* locus was determined by the distal genomic sequences, not the promoter per se. Mutations of two E-boxes and three E2F consensus sites at the proximal hTERT promoter region did not affect hTERT transcription in ESCs, but led to a stronger repression of the promoter during EB differentiation, indicating their roles in maintaining telomerase expression during differentiation. Thus, we demonstrated that the RMBT system in ESCs should facilitate the study of species-specific developmental regulation of human and mouse *TERT* genes.

## Results

### RMBT in ESCs

**Figure 1:**
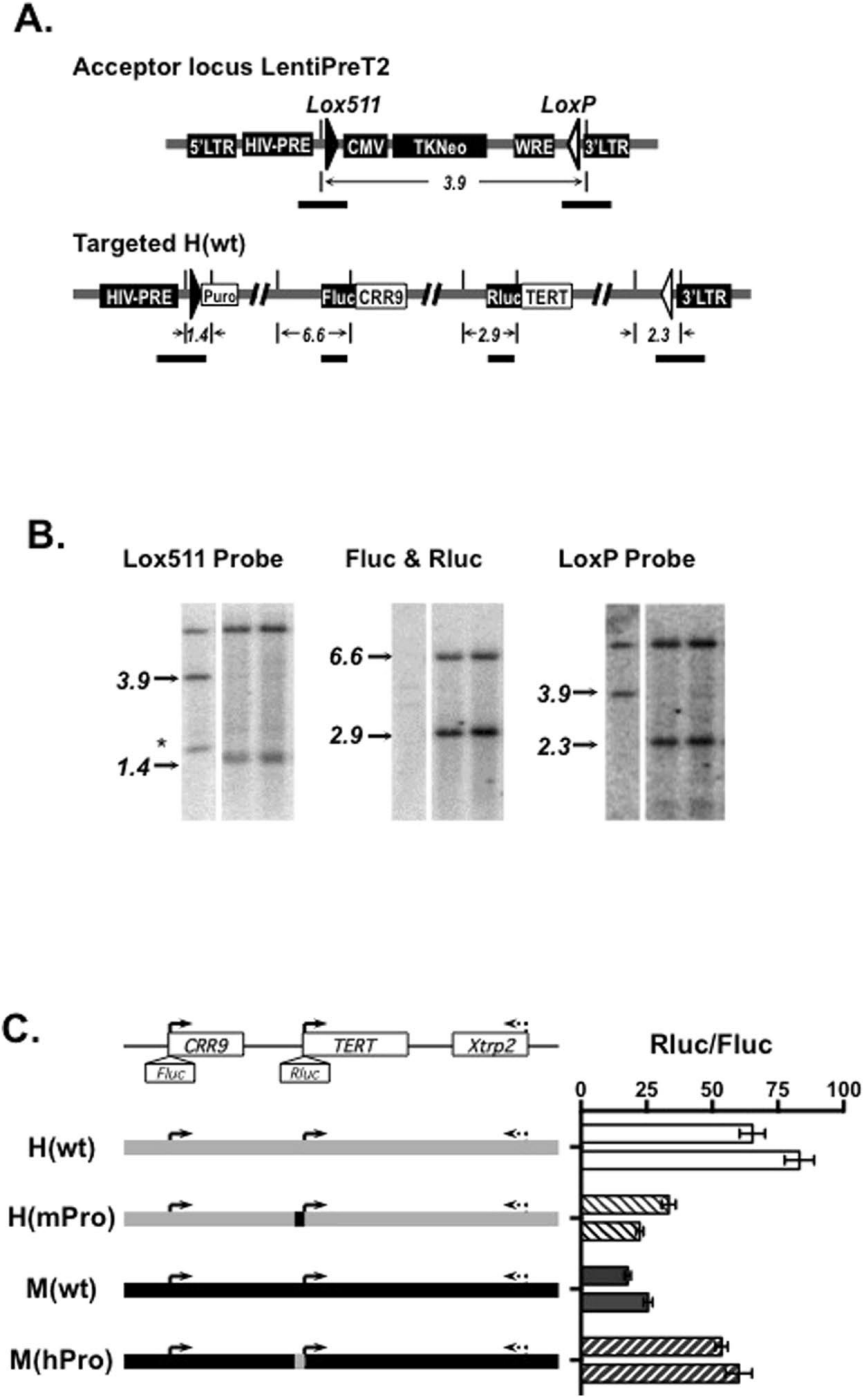
Chromosomal integration of BAC reporters. (**A**) Diagrams of chromosomal acceptor locus (top) and integrated BAC reporters (bottom). Lentiviral elements, selection markers, and luciferase reporters are shown as filled boxes. *Lox* sites are represented by triangles. Short vertical lines are *Dra* I sites and the numbers are sizes of restriction fragments in kilobases. Horizontal bars indicate positions of probes used in Southern blot analyses (see Table S1). (**B**) Southern analysis of RMBT clones. Genomic DNAs were extracted from acceptor cells (left lanes) and two clones containing H(wt) (middle and right lanes), digested with *Dra* I, and analyzed by Southern blotting using probes shown in A. Sizes of restriction fragments are indicated on the left of each panel. *, a band that was likely resulted from the presence of a *Dra* I site in a subset of the acceptor cells. The images were cropped from different parts of the same gel (see Supplementary info). (**C**) TERT promoter activities from BAC reporters in ESCs. Left, illustrations of wildtype and chimera BAC reporters. In H(wt) and M(wt), *Fluc* and *Rluc* cassettes were inserted at the initiation codons of *CRR9* and *TERT* genes, respectively. Grey and black lines represent human and mouse genomic sequences, respectively. In H(mPro) and M(hPro), the TERT promoters were swapped between H(wt) and M(wt). Right, luciferase activities were measured in cells from 96-well plates. The TERT promoter activities are shown as *Rluc*/*Fluc*. Two clones for each BAC reporter were analyzed.

Integration of BAC reporters into specific chromosomal sites by RMBT allows the analysis of genomic elements in a relevant genomic context^20^. To integrate BAC reporters in ESCs, we constructed a lentiviral vector pLentiPreT2 because the previously reported retroviral acceptor was silenced in pluripotent stem cells. The resulting proviral acceptor locus contained a tkNeo expression cassette, encoding a fusion protein of the HSV thymidine kinase and neomycin resistance genes (Fig. 1A, upper panel). This cassette was ‘floxed’ by *lox*511 and *lox*P sites that matched to the corresponding sites in BAC constructs. T2-5, a clone of TC1 cells containing a single provirus LentiPreT2 was used in subsequent experiments.

To directly compare *hTERT* and *mTERT* gene regulation during development, we first integrated H(wt) and M(wt), which contained genomic sequences encompassing the consecutive *CRR9*, *TERT*, and *Xtrp2* loci of the 160-bp human and 135-bp mouse genomic regions, respectively^21^, into T2-5 cells. Circular BAC DNAs and pCBM were co-transfected into T2-5 cells. Cre-mediated recombination between *lox*511/*lox*P sites on BACs and those of proviral LentiPreT2 resulted in the replacement of the tkNeo cassette by the entire BAC inserts. Individual clones were isolated following consecutive selection using puromycin and ganciclovir (GCV). Southern blot analyses were performed to determine the integrity of integrated BACs. For example, correct integration of H(wt) resulted in the loss of 3.9 kb *Dra*I band and the gain of a 1.4-kb and a 2.3-kb band at the *lox*511 and *lox*P site, respectively (Fig. 1B). The integrities of CRR9 and hTERT promoter regions were verified by the detection of a 6.6-kb and a 2.9-kb band using *Firefly* (*Fluc*) and *Renilla* (*Rluc*) luciferase probes, respectively. In addition to H(wt) and M(wt), we also generated ESC clones containing H(mPro) and M(hPro). H(mPro) and M(hPro) were resulted from swapping of the hTERT and mTERT promoters (472- and 474-bp genomic sequences immediately upstream of the hTERT and mTERT initiation codons, respectively) between H(wt) and M(wt)^21^. Thus, all these single-copy BAC reporters were integrated at the same chromosomal site and analyzed in the same chromatin environment.

### Regulation of TERT promoters during ESC differentiation

To determine the activities of TERT promoters in undifferentiated mouse ESCs, two independent ESC clones containing each BAC construct were analyzed (Fig. 1C). Comparing H(wt) with H(mPro) or M(hPro) with M(wt), the activities of hTERT promoter were 2–3 folds higher than those of mTERT promoter in the same genomic contexts. Comparing H(wt) to M(hPro) and H(mPro) to M(wt), hTERT and mTERT promoters were similarly active in human and mouse genomic contexts.

**Figure 2:**
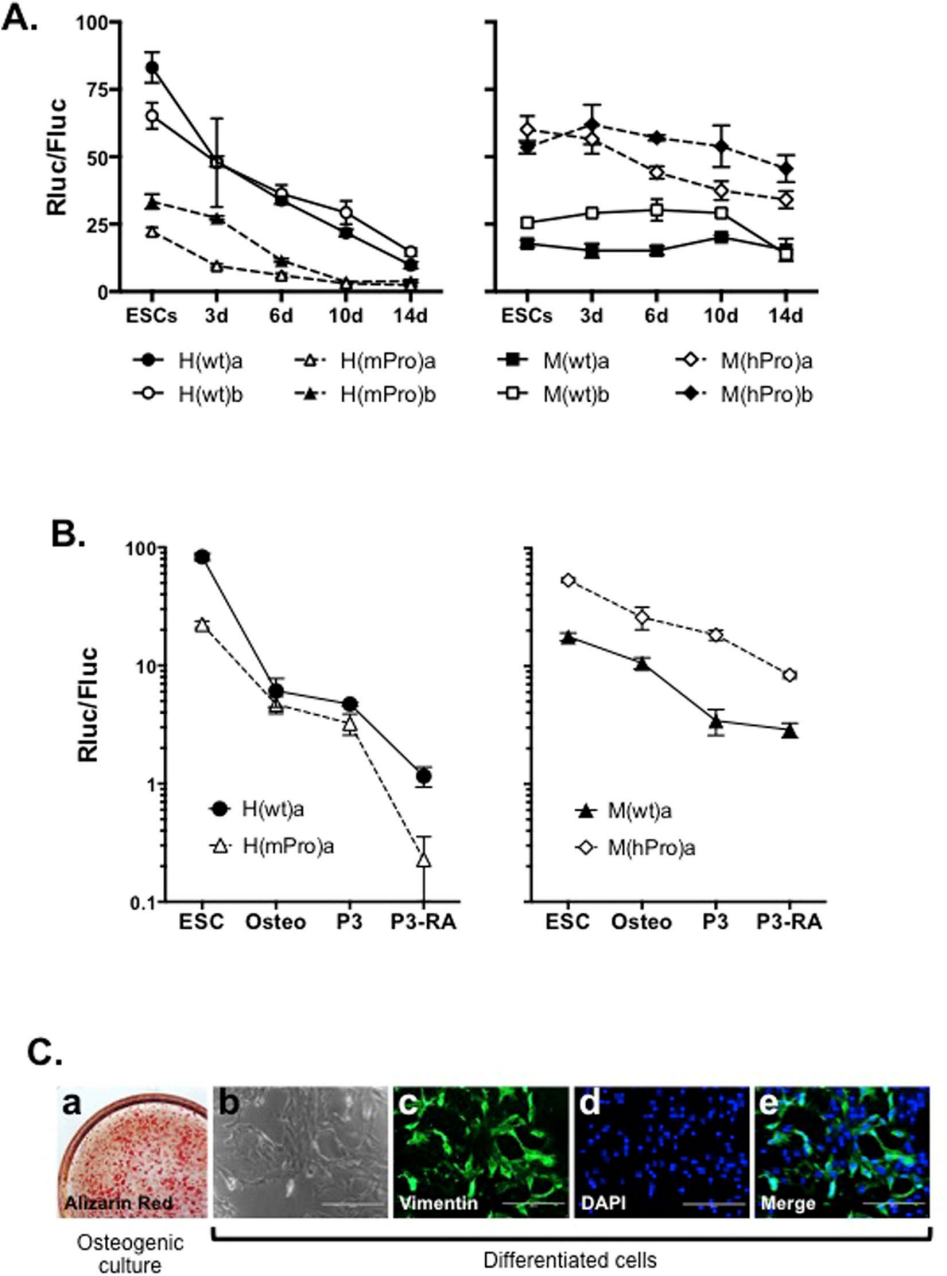
Regulation of TERT promoter activities during ESC differentiation. (**A**) TERT promoter activities in differentiating EB cultures. Cells were harvested for luciferase assays following the initiation of differentiation. (**B**) TERT promoter activities in differentiated osteogenic and fibroblast-like cells. Luciferase activities were measured in extracts from undifferentiated ESCs and their differentiated derivatives. P3, passage 3; P3-RA, P3 cells that were treated with 0.15 µM retinoic acid for two days. (**C**) Images of *in vitro* differentiated osteogenic and RA-treated cells. a, osteogenic cells were stained by Alizarin red. Differentiated cells were stained with an anti-vimentin antibody in right panels: b, bright field; c, vimentin antibody staining; d, DAPI staining; e, merged image.

Telomerase is expressed at very low levels in most human adult somatic tissues, but is readily detectable in most mouse tissues^13,15^. As an attempt to understand how *hTERT* and *mTERT* genes were regulated during development, ESCs were induced to differentiate into EBs for up to two weeks, and luciferase activities were determined during differentiation. As shown in Fig. 2A, the hTERT promoter in H(wt) was down-regulated steadily 5–10 folds over the course of 14-day differentiation. In contrast, the activity of mTERT promoter in M(wt) changed less than 2-fold over the same period. The regulation of TERT promoters in chimera BACs, H(mPro) and M(hPro), were also assessed. Although the activity of mTERT promoter of H(mPro) was lower than that of the hTERT promoter in H(wt) in undifferentiated ESCs, its activity was reduced by 8–10 folds during EB differentiation, similar to the hTERT promoter in H(wt). Conversely, the activity of hTERT promoter in M(hPro) was maintained during the process, similar to the mTERT promoter in M(wt). Therefore, the strong repression of the hTERT promoter during EB differentiation was determined by its genomic environment and the mouse genomic context did not have strong repressive effect.

We previously reported that the hTERT promoter was further repressed during differentiation into cells of more specific lineages^13^. Thus, EBs were induced to differentiate into the osteogenic lineage, and the resulting cells were subsequently cultured in 10% fetal bovine serum and became morphologically fibroblast-like cells (Fig. 2C). Many of these cells expressed vimentin, a mesenchymal marker found in fibroblasts. Treatment of these cells for two days with retinoic acid further increased the expression of this marker. As shown in Fig. 2B, both hTERT and mTERT promoters in the human genomic context, H(wt) and H(mPro), were repressed about 100-fold during this differentiation process. As a comparison, the TERT promoters in the mouse genomic context, M(wt) and M(hPro), were down-regulated by about 6-fold. Hence, while the hTERT promoter was stronger than the mTERT promoter, genomic contexts play a dominant role in determining their repression during differentiation.

### Epigenetic states of TERT loci in ESCs and differentiated cells

**Figure 3:**
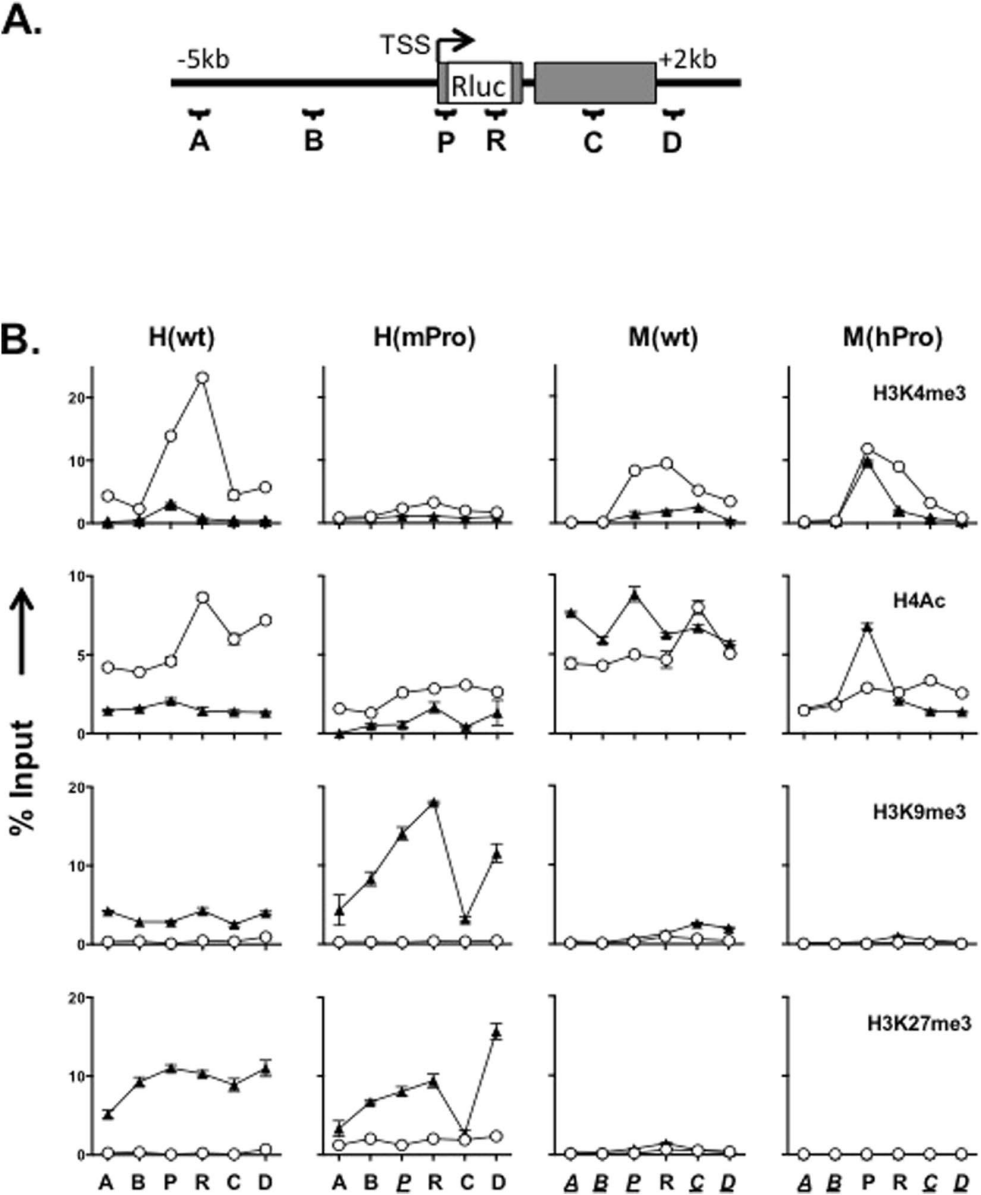
Chromatin structures of *TERT* genes in ESCs and differentiated cells. (**A**) A schematic diagram of the genomic region spanning the TERT promoters in the BAC reporters. Positions of qPCR amplicons are labeled as: A, −5 kb; B, −2 kb; P, TERT promoter; R, *Rluc* ORF (approximately + 1 kb downstream of the TERT promoters in the BAC reporters); C, + 1 kb; and D, +2 kb. The positions are relative to the TERT TSSs in the native genomic sequence (without *Rluc* ORF). Italic/underlined labels indicate corresponding mouse amplicons. (**B**) Covalent histone modifications at the TERT promoters. Chromatin fragments from ESCs containing the BAC reporters were precipitated using antibodies against specific histone marks, followed by qPCR analyses. H4Ac refers to acetylated histone H4. H3K4me3, H3K9me3, and H3K27me3 are trimethylated K4, K9, and K27 residues of histone H3. Open circles and closed triangles are undifferentiated ESCs and differentiated fibroblast-like cells, respectively.

To determine epigenetic changes associated with developmental regulation of the *TERT* genes, we examined an 8-kb region around the TERT promoters in the BAC reporters for a representative set of covalent histone modifications using chromatin immunoprecititation (ChIP) and quantitative PCR analysis (Fig. 3). Positive histone marks H3K4me3 and H4Ac were highly enriched at the hTERT promoter in H(wt) in ESCs and their levels decreased significantly in differentiated cells (Fig. 3B). Both H3K4me3 and H4Ac at the mTERT promoter in H(mPro) were lower than those of the hTERT promoter in H(wt) in ESCs, consistent with the data that the mTERT promoter was less active than the hTERT promoter. Both marks also decreased upon differentiation. Likewise, relatively high levels of H4Ac and H3K4me3 were also detected at the TERT promoters in M(wt) and M(hPro) in ESCs. H4Ac levels did not decline radically upon differentiation. However, H3K4me3 decreased at the mTERT promoter in M(wt) but remained at the hTERT promoter in M(hPro). Overall, the levels of H3K4me3 and H4Ac correlated with the TERT promoter activities in ESCs and differentiated cells.

H3K9me3 and H3K27me3, are two epigenetic marks associated with gene silencing. In ESCs, the hTERT and mTERT promoters in H(wt) and H(mPro) contained little H3K9me3 and H3K27me3. These marks dramatically increased in differentiated cells. However, no H3K9me3 and H3K27me3 were detected at M(wt) and M(hPro) in either ESCs or their differentiated derivatives. These results suggested that the human genomic context conferred a repressive chromatin environment and led to a stronger repression of the *hTERT* locus during differentiation. This repression was accompanied by dramatic increases of both H3K9me3 and H3K27me3 in differentiated cells.

### Chemical inhibition of histone deacetylases (HDACs)

**Figure 4:**
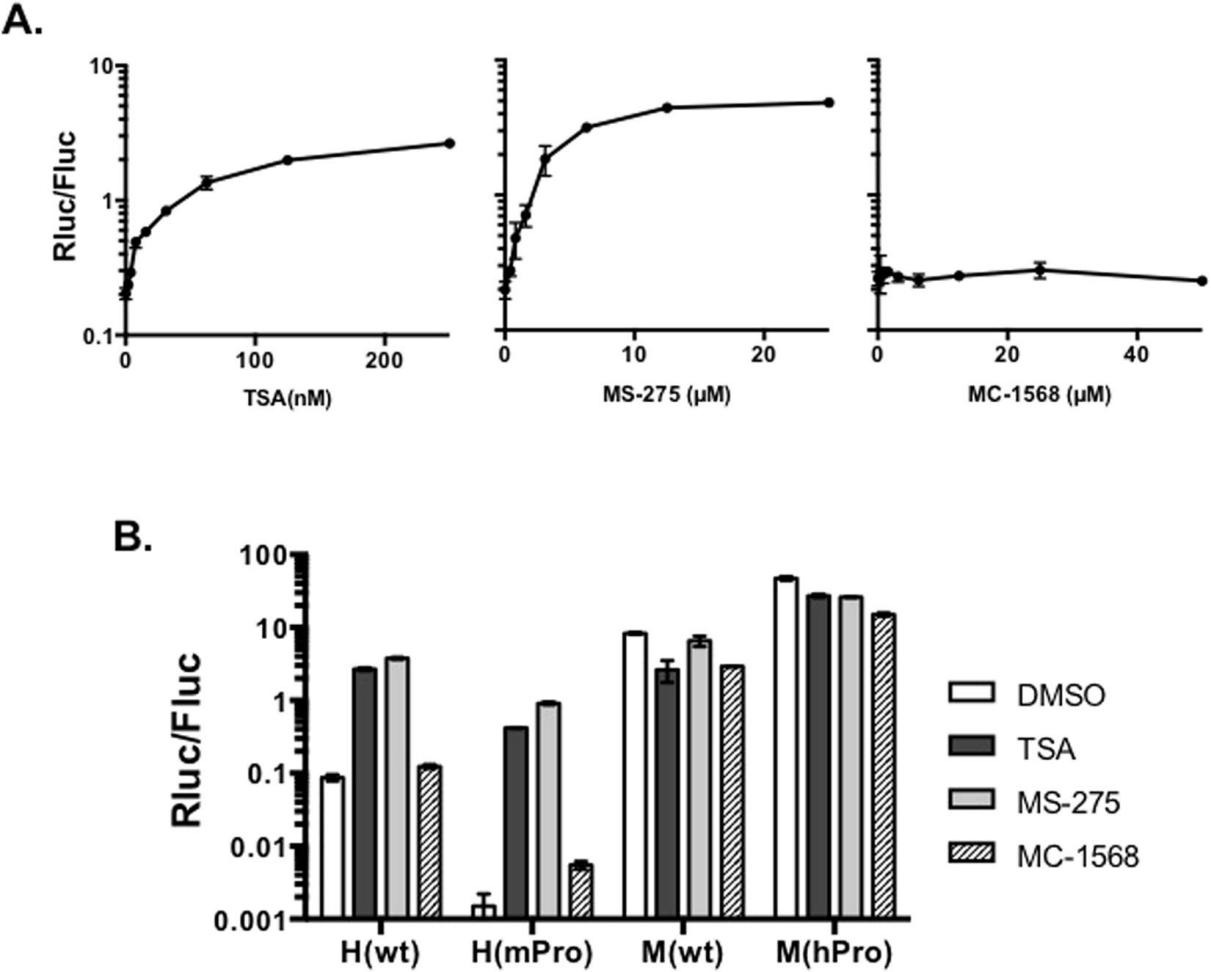
Induction of TERT promoters in the BAC reporters by HDAC inhibitors in differentiated cells. (**A**) Differentiated cells containing H(wt), H(wt)a, were treated with increasing amounts of HDAC inhibitors for 24h. (**B**) Differentiated cells derived from ESC clones H(wt)a, H(mPro)a, M(wt)a, and M(hPro)a were treated with 250 nm TSA, 25 µM MS-275, or 50 µM MC-1568 for 24 h. hTERT promoter activity was determined as *Rluc*/*Fluc*.

Recently, we reported that hTERT repression in immortal human fibroblasts involved multiple HDAC1-containing corepressor complexes (CRCs)^21^. To verify that HDACs were also involved in hTERT repression during ESC differentiation, differentiated mouse cells containing H(wt) were treated with chemical inhibitors, trichostatin A (TSA, an inhibitor of both classes I & II HDACs), MS-275 (a specific inhibitor of class I HDACs), and MC-1568 (an inhibitor of class II HDACs). As shown in Fig. 4A, the hTERT promoter in H(wt) was activated by TSA and MS-275, but not MC-1568, in a dose-dependent manner. In cells containing H(mPro), the mTERT promoter was similarly induced by 250 mM TSA and 25 mM MS-275 (Fig. 4B). However, MC-1568 had little effect on the hTERT promoter in H(wt) and induced the mTERT promoter in H(mPro) only 3-fold. On the other hand, none of these inhibitors induced TERT promoters in the mouse genomic context. Lastly, the mTERT promoter was more stringently repressed than the hTERT promoter in the human genomic context, consistent with that the hTERT promoter was a stronger promoter. Thus, both TERT promoters were subjected to strong repression, mediated mainly by class I HDACs, in the human, but not mouse, genomic context.

### Association of transcription factors (TFs) to TERT promoters

**Figure 5:**
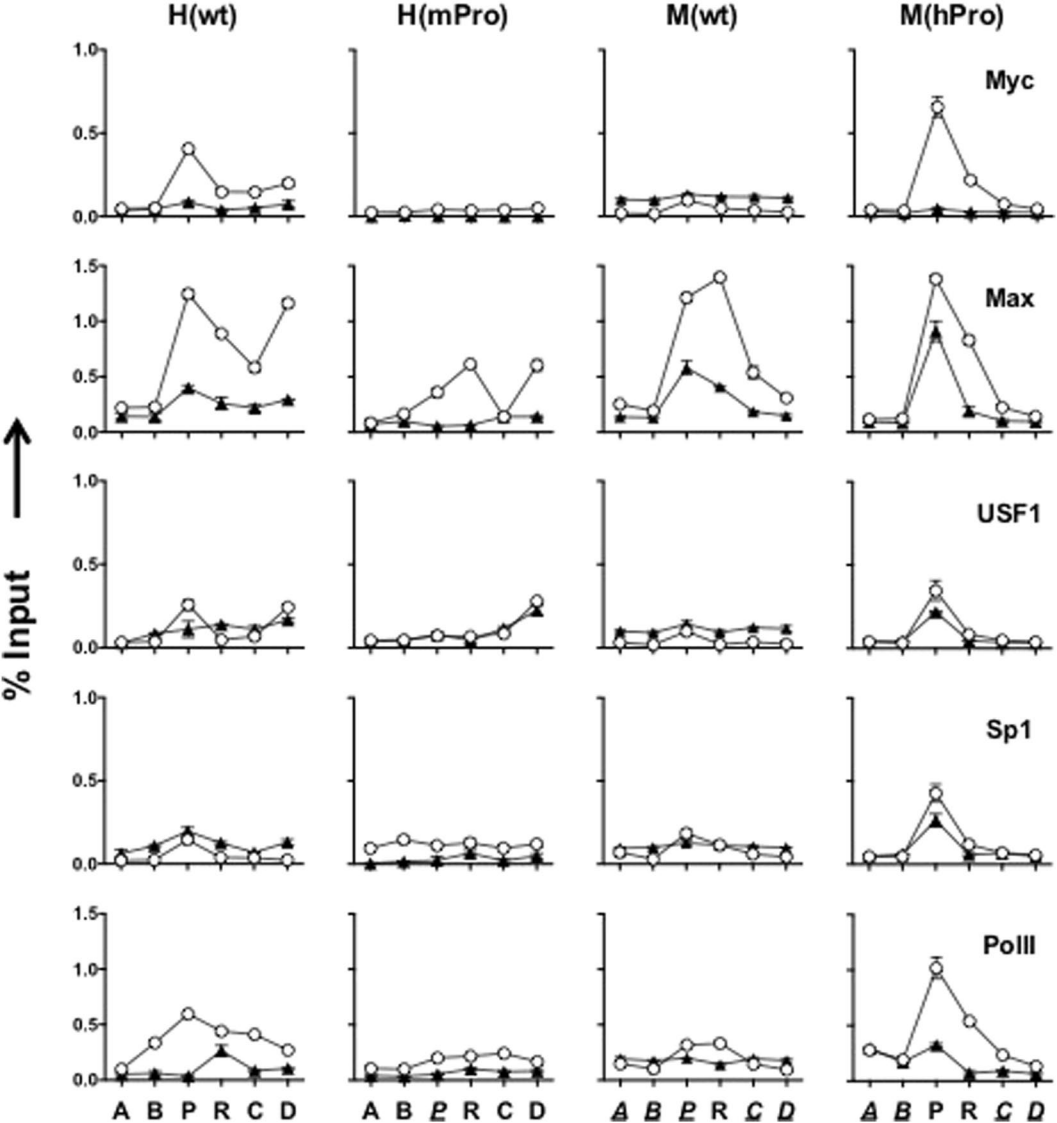
Binding of TFs at the TERT promoters. ChIP experiments were performed as in Fig. 3B using antibodies against TFs. Open circles and closed triangles are undifferentiated ESCs and differentiated fibroblast-like cells, respectively.

Next, we assessed the binding of several TFs to the TERT promoters. Proto-oncogene product c-Myc and its heterodimer partner Max were known to bind to the E-boxes at the hTERT promoter and activate hTERT transcription in cancer cells^22^. As shown in Fig. 5, both c-Myc and Max proteins bound to the hTERT promoter in H(wt) and M(hPro) in ESCs and their binding decreased in differentiated cells. However, substantial Max binding remained at the hTERT promoter in M(hPro) after differentiation. Max, but not c-Myc, bound to the mTERT promoter region in M(wt) and H(mPro) and its binding decreased in during differentiation. The reason why Max, but not c-Myc, bound to the hTERT promoter in differentiated cells and to the mTERT promoter is unclear at the present time. The Max antibody used in this study was more sensitive than all c-Myc antibodies we had tested and might have contributed to the stronger Max signal. Alternatively, Max might bind to the promoter as a homodimer, or a heterodimer with other c-Myc family proteins, such as Mxd1, especially in differentiated cells^23^. The binding of USF1, also an E-box binding TF, and Sp1 was relatively weak at the TERT promoters. Finally, the presence of PolII at the TERT promoters correlated with their activities in ESCs and this association decreased in all BAC reporters in differentiated cells. Overall, the association of TFs was consistent with the regulation of TERT promoters in both ESCs and differentiated cells.

### Roles of TF binding at the hTERT promoter

**Figure 6:**
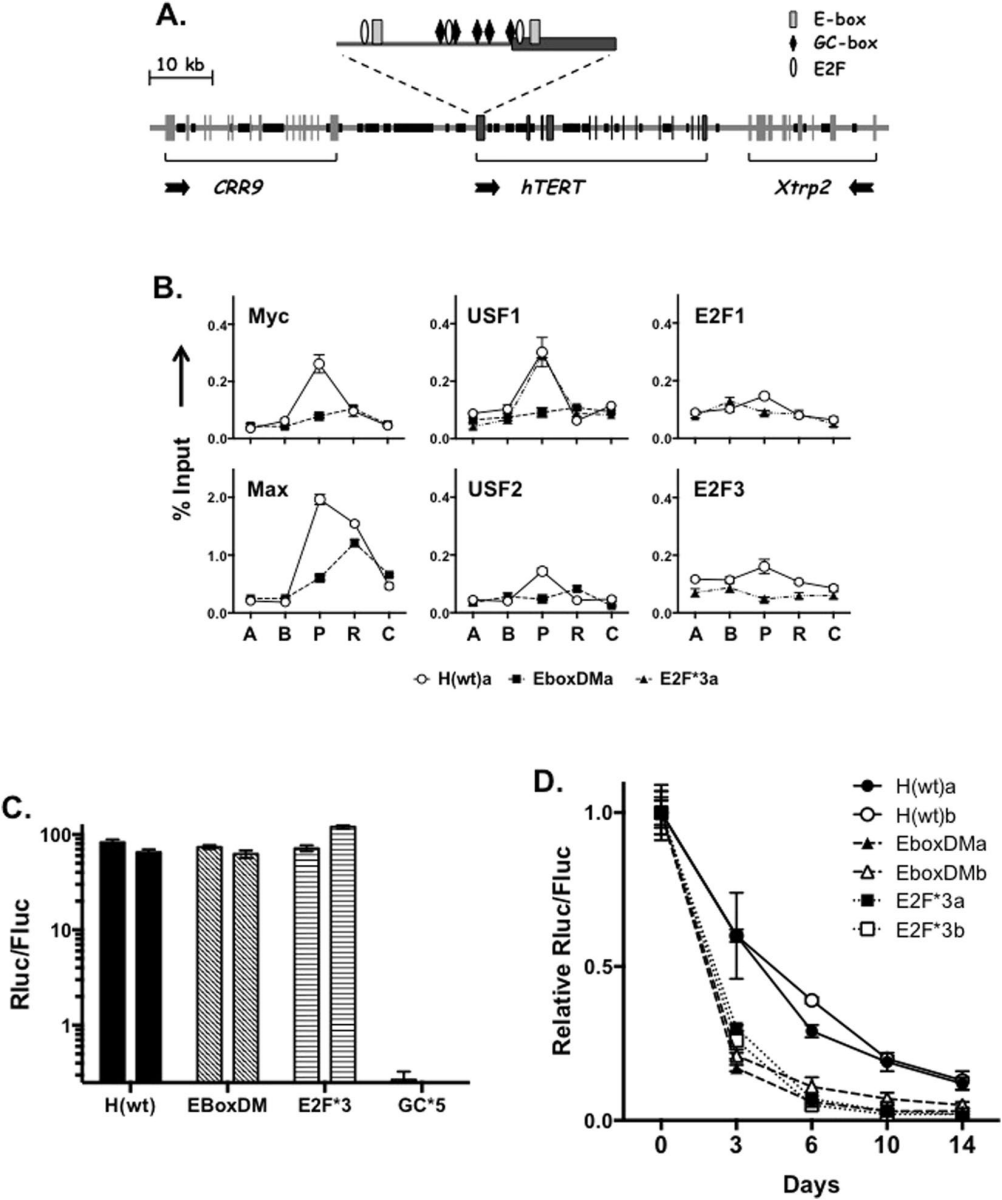
Roles of TF binding sites on hTERT promoter activity in ESCs and during *in vitro* differentiation. (**A**) A schematic illustration of TF binding sites at the hTERT promoter in BAC reporter H(wt). Vertical bars and lines represent exons. Black regions of horizontal lines are repetitive sequences; horizontal arrows indicate directions of transcription. Expanded above is the hTERT promoter region upstream of ATG codon, in which the thick region represents sequence of the first hTERT exon. (**B**) TF binding to the hTERT promoter. Chromatin fragments from ESCs containing H(wt), H(EboxDM), and H(E2F*3) were precipitated using antibodies against TFs as indicated and ChIP experiments were performed as in Fig. 5. (**C**) Activities of mutant hTERT promoters in ESCs. Luciferase activities were determined in proliferating ESCs containing point mutations that eliminated two E-boxes (EboxDM), three E2F consensus sites (E2F*3), and five GC-boxes (GC*5). Two independent clones of each mutant BAC were tested. (**D**) Activities of mutant hTERT promoters during EB differentiation. Luciferase activities were measured in differentiating EB cultures.

It has been reported that multiple TFs bound to their cognate sites at the hTERT promoter and regulated its activity. These sites included E-boxes, E2F sites, and GC-boxes that are binding sites for TFs of basic helix-loop-helix family, E2F family, and Sp1 family, respectively^24–26^. However, most previous studies were done in the contexts of transiently transfected small reporter plasmids. To determine how these TFs regulated hTERT transcription in ESCs and during differentiation, point mutations were introduced to several TF sites in the BAC reporter H(wt). These sites were two E-boxes, three E2F consensus sites, and five GC-boxes at the hTERT promoter (Fig. 6A). The mutant reporters were integrated into the same acceptor site in T2–5 cells and two independent clones for each reporter were analyzed. Mutation of two E-boxes reduced the binding of c-Myc, Max, USF1, and USF2 to the hTERT promoter in H(EboxDM) (Fig. 6B). The binding signals for E2F1 and E2F3 at the hTERT promoter in ESCs were relatively weak, but they were reproducibly lower at the mutant promoter in H(E2F*3) than those at the wildtype promoter in H(wt). In addition, USF1 binding to H(E2F*3) was normal, indicating that its binding was independent of E2F factors. Interestingly, mutant hTERT promoters in H(EboxDM) and H(E2F*3) were as active as the wildtype promoter in H(wt) in ESCs (Fig. 6C). On the other hand, elimination of five GC-boxes in H(GC*5) completely abolished hTERT transcriptional activity. These results indicated that, while the GC-boxes were critical promoter elements, the E-boxes and E2F sites were not essential for the hTERT promoter function in undifferentiated ESCs.

To assess the roles of TF binding sites during differentiation, ESCs containing BAC reporters were induced to differentiate in EB cultures. As shown in Fig. 6D, transcription from the hTERT promoter in H(wt) decreased 30–40% in the first 3 days and about 5-fold over two weeks. The activity of mutant hTERT promoter in H(EboxDM) rapidly decreased 5–6 folds within 3 days and 20–30 folds in two weeks. Similarly, the mutant hTERT promoter in H(E2F*3) were down-regulated 3–4 folds in 3 days and about 50-fold after two weeks. Thus, while loss of E-box-binding proteins and E2F family proteins did not affect hTERT promoter activity in ESCs, these proteins were important for maintaining hTERT transcription during differentiation.

## Discussion

Telomerase is highly expressed in pluripotent stem cells and is required for their immortal proliferation, but the mechanisms of TERT gene regulation in these cells remain to be determined. In this study, we report the development of RMBT technique in ESCs to investigate of hTERT and mTERT gene regulation in ESCs and during differentiation.

Previously, we used the RMBT technique to study hTERT regulation in immortal human fibroblasts^20,21^. In those experiments, the *de novo* chromatin around BAC reporters was assembled from transfected naked BAC DNAs without going through the same stepwise epigenetic modifications as those of endogenous loci^19,27^. Yet, the integrated BAC reporters in ESCs are likely subjected to similar epigenetic regulation during differentiation. Thus, the RMBT method in ESCs is especially suitable for studying genetic elements required for developmental gene regulation. First, it involves integration of large single-copy BAC reporters (up to 300-kb) into an acceptor site and allows the studies of distal elements in correct genomic contexts. Since BACs are not integrated at the endogenous locus, the *mTERT* gene in ESCs remains intact and its regulation is unlikely affected by manipulation of the reporters. This technique should also be applicable for studying genes essential for stem cell proliferation and differentiation. Second, because human and mouse genomic sequences bear little resemblance outside of their coding regions, the endogenous *mTERT* locus does not interfere with the epigenetic studies of the human sequences, such as ChIP-qPCR. Third, BAC recombineering allows more precise and complex manipulation of BAC DNA sequences than the genomic editing techniques using the CRISPR-Cas9^28–30^. Hence, the RMBT method combined with BAC recombineering will still provide a useful tool for analysis of genomic elements in the foreseeable future. Fourth, the use of sensitive bioluminescent reporters allows the detection of relatively small changes in transcriptional regulation. Finally, the RMBT technique is used to analyze human genes in mouse ESCs which are easier to culture and manipulate than human pluripotent stem cells^31^.

In this first study using RMBT in ESCs, our results corroborated previous reports that the *hTERT* and *mTERT* genes were highly expressed and not repressed in undifferentiated ESCs^13,19,27^. We also showed that the repressive chromatin of the *hTERT* gene was set up progressively starting early EB differentiation. A significant decrease of hTERT transcription was readily detected within 3 days after the initiation of differentiation. The repression was accompanied by marked increases of both H3K9me3 and H3K27me3 at the hTERT promoter. In contrast, the mTERT promoter in M(wt) remained active throughout EB differentiation, and its activity reduced moderately only following osteogenic differentiation. No detectable H3K9me3 and H3K27me3 marks were found at the mTERT promoter in ESCs or differentiated cells. Importantly, the human-specific repression during differentiation was determined by distal genomic sequences, not the promoters, as indicated by data from chimera BACs H(mPro) and M(hPro). This result was also consistent with our previous report that hTERT repression in human fibroblasts required the human genomic context using the same set of BAC reporters^21^. Determination of sequence requirement for the hTERT repression should help to identify *cis* elements required for repression. These distal elements likely recruit class I HDACs CRCs to the promoter and mediated the repression.

Comparing human and mouse sequences, one notable difference is that human 5′ intergenic region (5’IR, 23-kb) is much larger than its mouse counterpart (7-kb) (Fig. 6A). This difference is largely due to the presence of abundant repetitive sequences in human, but not mouse, 5’IR. The repetitive sequence, making up 50% of human 5’IR, contains mostly transposable elements (TEs), such as LINEs, SINEs, and LTR retrotransposons. In addition to TEs, the *hTERT*, but not *mTERT*, gene also contains five minisatellites (also called variable number of tandem repeats), in introns 2, 6, and 12. It is conceivable that these repetitive elements help to create a repressive chromatin environment at the *hTERT* locus. Based on published studies, it is tempting to speculate that two mechanisms contribute to transcriptional repression. First, these elements may function as silencers by binding to repressors. For example, TEs have been shown to recruit polycomb group (PcG) repressor complexes^32^ and KAP1/NuRD repressor complexes via KRAB family zinc finger proteins (KRAB-ZNFs)^33^, leading to the accumulation of H3K27me3 and H3K9me3, respectively. Second, although contradictory, transcription may occur in non-coding regions containing repetitive sequences^34^. The resulting transcripts may be aberrantly processed and recognized through base-pairing by small interfering RNAs, which recruit complexes containing Argonaute proteins, leading to histone modifications and the formation of heterochromatin^35,36^. The RNAi-mediated transcriptional gene silencing has been well studied in model organisms, but its mechanisms in mammals are only beginning to be understood^37,38^.

The highest hTERT transcription is found in pluripotent stem cells^13,19,39^. How this expression is maintained in these cells remains to be elucidated. Our data showed that elimination of E-boxes and E2F consensus sites, binding sites for two critical TF families, had little effect on hTERT transcription in ESCs, indicating that these TFs were not required for hTERT promoter activity in these cells. It was possible that redundant TFs activated the hTERT promoter in ESCs. Alternatively, the chromatin of *hTERT* locus might be wide-open in ESCs^40^ and transcriptional machineries could be easily loaded onto the promoter without the facilitation by TFs. Interestingly, the mutant BAC reporters lacking either E-boxes or E2F sites underwent rapid down-regulation during EB differentiation, more quickly than H(wt), suggesting that TF bound to these sites played important roles in maintaining hTERT transcription in differentiating cells. Upon differentiation, the chromatin around the hTERT promoter began to condense and it was critical for TFs to bind to the promoter and maintain its expression during differentiation. Based on this result, it would be interesting to determine whether these TFs were required for hTERT transcription in adult stem cells and progenitor cells.

The regulation of *hTERT* gene is very complex. It was recently reported that the *hTERT* gene might also be regulated by telomeric position effect via telomere looping at the end of chromosome 5p^41–43^. The 160-kb human sequence in H(wt) did not contain any interstitial telomeric sequence of (TTAGGG)_2_ or longer and the reporter nonetheless recapitulated endogenous hTERT regulation in an integration site-independent manner in both ESCs cells and transgenic mice^13,15,20^. These results suggested that H(wt) contained sufficient regulatory sequence for hTERT regulation. In addition to transcriptional regulation, hTERT mRNA was also subjected to alternative splicing^44,45^. In H(wt), a *Renilla* luciferase cassette was inserted at the ATG codon in the first hTERT exon, and the reporter was unlikely affected by splicing. Despite of these potential shortcomings, H(wt) recapitulated endogenous hTERT regulation during mouse development and ESC differentiation^13,15^ and should provide valuable insights into the mechanisms of its regulation.

In summary, we have developed a system to study the transcriptional regulation of *hTERT* gene in mouse ESCs. Whereas epigenetic mechanisms have been linked to gene silencing, relatively little is known about how epigenetic memories are directed to target DNA sequences during development and in response to extracellular environment. The identification of *cis* elements is important for elucidating the mechanisms of not only hTERT repression in most somatic cells, but also its tissue-specific expression in thymus, skin, testis, and ovary, where telomerase is critical for T cell functions, skin regeneration, and germ cell proliferation and maintenance. Finally, this technical platform should also be applicable to the investigation of other genes and genomic regions.

## Methods

### BAC Reporters

BAC reporters H(wt), M(wt), H(mPro), and M(hPro) were described previously^20,21^. The two E-box sites (−165 and +43nt, relative to the transcription start site, TSS), three E2F sites (−174, −98, and +10nt), and five GC-boxes (−110, −90, −58, −36, & −9 nt) at hTERT core promoter in H(wt) were mutated via BAC recombineering^28^, resulting in H(EboxDM), H(E2F*3), and H(GC*5), respectively. H(EboxDM) and H(GC*5) were reported previously^22^. The E2F consensus sites in H(E2F*3) were mutated as (−174)CGCGC→CATGC, (−98)CGCGC→CATGC, and (−10)GCGCG→GCATA. All mutations were verified by sequencing.

### Cell culture, RMBT, and differentiation

Mouse ESC line TC1^46^ was cultured as previous described^13,19^. TC1 cells were infected with a low titer lentivirus LentiPreT2 and neomycin-resistant clones were isolated. T2–5, a clone with a single-copy provirus, was cotransfected with BAC reporters and a Cre-expressing plasmid pCBM^20^. Following consecutive selections with puromycin and GCV, clones with both *Fluc* and *Rluc* activities were isolated and analyzed. Southern analysis of RMBT clones was performed^20^ using probes listed in Table S1.

Differentiation of ESCs into EBs and osteogenic cultures were described previously^13^. On days 0, 3, 6, 10 and 14, individual EBs were harvested for luciferase assay. Differentiated osteogenic cells were analyzed by Alizarin red staining^47^. To promote fibroblast-like differentiation, passaged cells were treated with 0.15 *µ*M retinoic acid (Sigma-Aldrich, St. Louis, MO, USA) for 2 days. Fibroblast-like cells were characterized by Immunofluorescence staining using vimentin antibody (Thermo Fisher Scientific, Waltham, MA, USA).

### Gene expression analyses

Luciferase activities were measured using Dual-Luciferase® Reporter Assay System (Promega, Madison, WI, USA). As both hCRR9 and mCRR9 genes were constitutively expressed and their expression did not change during differentiation^48^, *Rluc* activities from the TERT promoters were normalized to *Fluc* activity from the CRR9 promoters. Moreover, all data were verified by normalizing *Rluc* activity to the number of cells, as determined by thiazolyl blue tetrazolium bromide (MTT) assays (data not shown). To induce hTERT transcription, differentiated cells were treated with HDAC inhibitors TSA, MS-275, or MC-1568 for 24 h and luciferase activities were determined. All reporter assays were performed in triplicate and repeated at least once.

### Chromatin Immunoprecipitation (ChIP)

ChIP assay was done as in previous publications^21,22^. The precipitated chromatin DNA fragments were analyzed by quantitative PCR. Antibodies are listed in Tables S2.

The datasets generated during and/or analysed during the current study are available from the corresponding author on reasonable requests.

## Electronic supplementary material

Supplementary Information
