# Supplementary material for: Regulation of human and mouse telomerase genes by genomic contexts and transcription factors during embryonic stem cell differentiation
Source: Sci Rep. 2017 Nov 27;7:16444. doi: 10.1038/s41598-017-16764-w (PMC5703907; doi:10.1038/s41598-017-16764-w)

Regulation of human and mouse telomerase genes by genomic contexts and transcription factors during embryonic stem cell differentiation

De Cheng[1,*], Shuwen Wang[1], Wenwen Jia[2], Yuanjun Zhao[3], Fan Zhang[1], Jiuhong, Kang[2], and Jiyue Zhu[1,*]

Supplementary Information

Table S1. Southern blot probes

Probes	Plasmids	Restriction fragments	Fragment size
**Fluc**	pYF10	Sph I-Xba I	989 bp
**Rluc**	pYF2-hRluc	Nhe I-Xba I	945 bp
**Lox511 junction**	pLentiPreT2	Hind III-Sac II	1199 bp
**LoxP junction**	pLentiPreT2	Hind III-PflM I	953 bp

Table S2. Antibodies

Antibodies	Sources	Cat. #	Manufactures	Applications
anti-c-Myc	Rabbit polyclonal	sc-764X	Santa Cruz Biotechnology	ChIP
anti-Max	Rabbit polyclonal	sc-197	Santa Cruz Biotechnology	ChIP
anti-USF1	Rabbit polyclonal	sc-229X	Santa Cruz Biotechnology	ChIP
anti-Sp1	Rabbit polyclonal	07-645	EMD Millipore	ChIP
anti-E2F1	Rabbit polyclonal	sc-193	Santa Cruz Biotechnology	ChIP
anti-E2F3	Rabbit polyclonal	sc-878	Santa Cruz Biotechnology	ChIP
anti-H4Ac	Rabbit polyclonal	06-866	EMD Millipore	ChIP
anti-H3K4me3	Rabbit polyclonal	07-473	EMD Millipore	ChIP
anti-H3K9me3	Rabbit polyclonal	ab8898	Abcam	ChIP
anti-H3K27me3	Rabbit polyclonal	07-449	EMD Millipore	ChIP
Normal rabbit IgG	Rabbit	sc-2027	Santa Cruz Biotechnology	ChIP
Anti-Vimentin	Mouse monoclonal	MA5-11883	Themo Fisher Scientific	IF

Fig 1B Lox511

Scanner Typhoon 9400

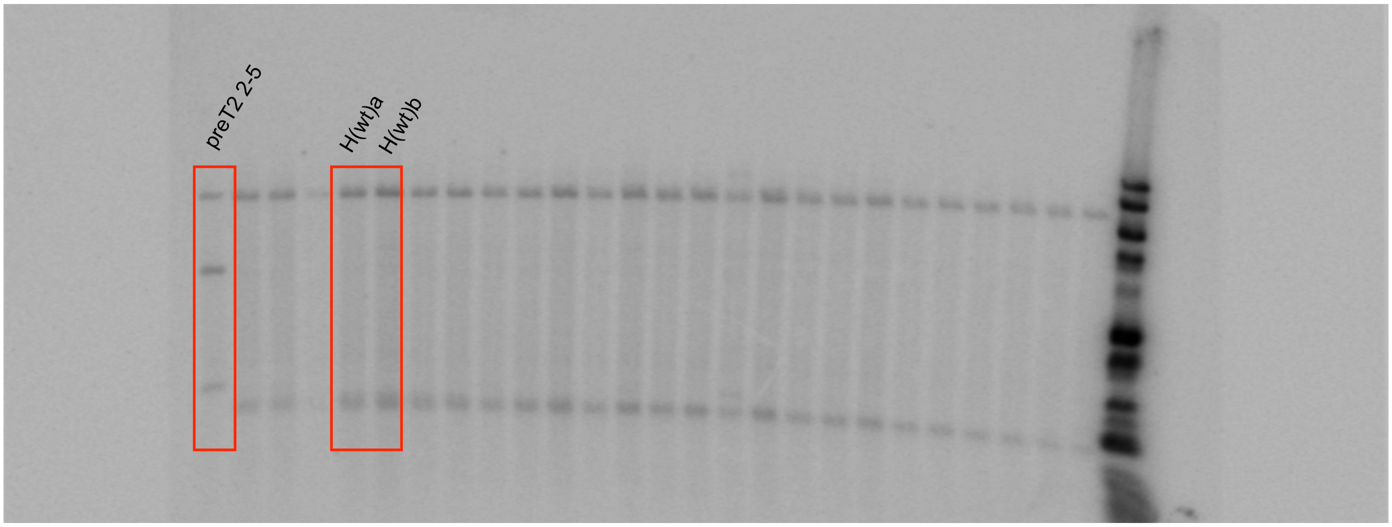

Fig 1B Fluc&Rluc

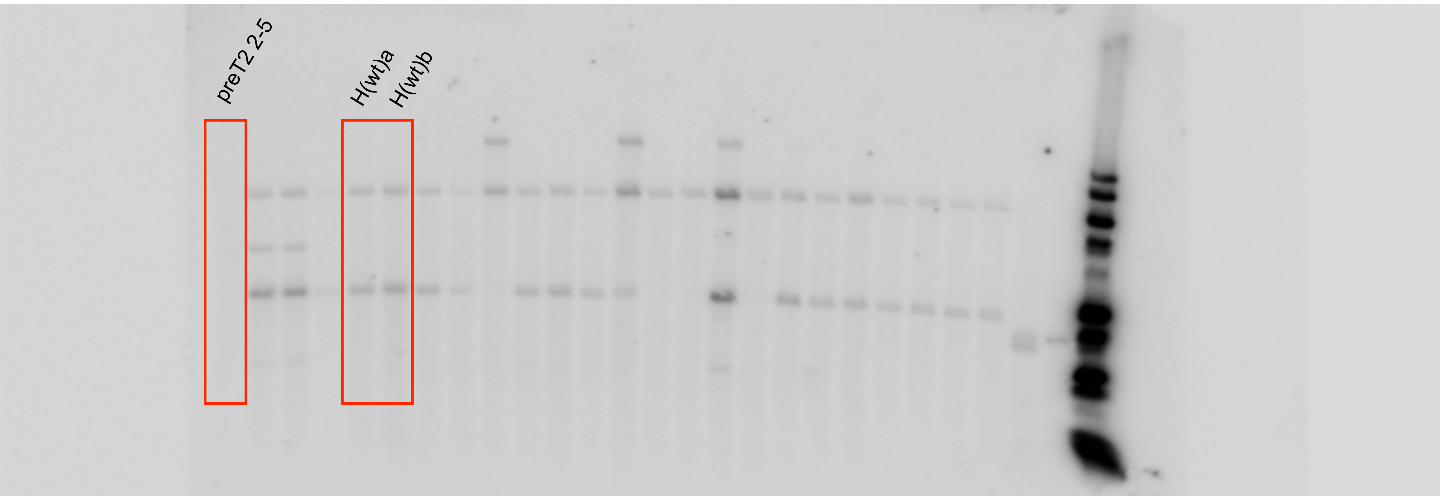

Fig 1B LoxP Probe

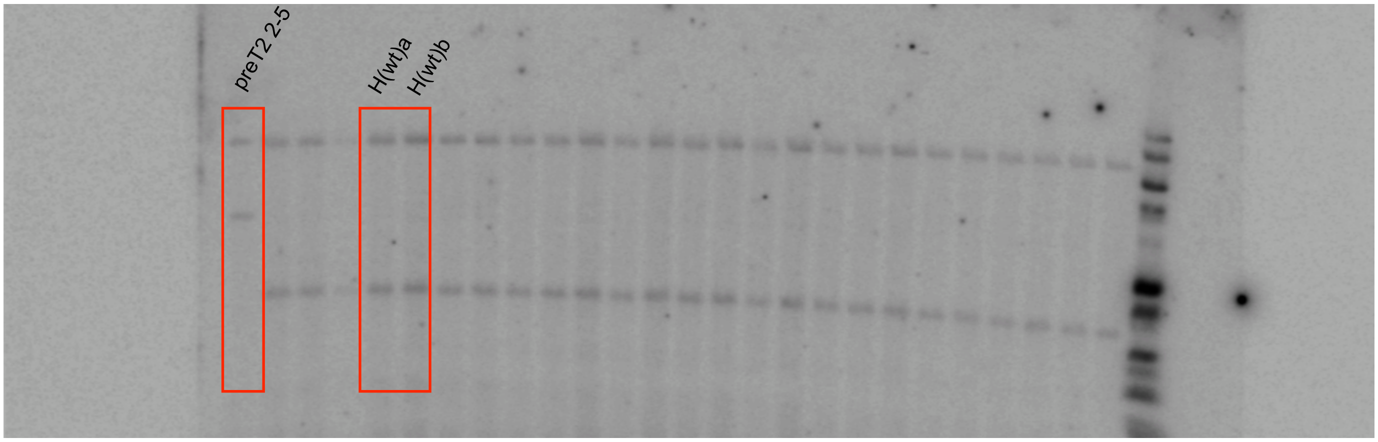

Supplement: Supplementary file 1 — Supplementary Information [file 41598_2017_16764_MOESM1_ESM.pdf]
